# Initiation of multiple-session psychological care in civilians exposed to the November 2015 Paris terrorist attacks

**DOI:** 10.1186/s13690-023-01206-z

**Published:** 2023-11-29

**Authors:** Philippe Pirard, Yvon Motreff, Lise Eilin Stene, Gabrielle Rabet, Cécile Vuillermoz, Stéphanie Vandentorren, Thierry Baubet, Antoine Messiah

**Affiliations:** 1 Santé Publique France, French National Public Health Agency, Saint-Maurice, F-94415 France; 2 Team MOODS, Inserm-CESP, Université Paris-Saclay, UVSQ, 94807 Villejuif, France; 3 Department of Social Epidemiology, INSERM, Sorbonne Université, Institut Pierre Louis d’Epidémiologie et de Santé Publique, Paris, F75012 France; 4 Norwegian Centre for Violence and Traumatic Stress Studies (NKVTS), Oslo, Norway; 5 UMR 1219, Bordeaux Population Health Research Center, PHARes Team, University of Bordeaux, Bordeaux, France; 6 Université Sorbonne Paris Nord, UTRPP EA 4403, Villetaneuse, France; 7 AP-HP, Hôpital Avicenne, Bobigny, France; 8 Resources and Resilience National Centre (CN2R), LilleParis, France

**Keywords:** Terrorist attacks, Psychological care, Post-traumatic stress disorder, Mental health treatment, Psychological first aid

## Abstract

**Background:**

Terrorist attacks can induce post-traumatic stress disorder (PTSD) and depression, which require multiple-session psychological care (MSPC). This study aims at investigating MSPC initiation and associated factors.

**Methods:**

Data were collected from a web-based survey of civilians 8–12 months after their exposure to the November 2015 Paris terrorist attacks. Depression and partial and full PTSD were assessed using the Hospital Anxiety and Depression Scale and the PCL-5 checklist, respectively. Questionnaires collected data on socio-demographic variables, exposure to the attacks, psychological treatment history, social isolation, somatic problems, having received an outreach psychological support (OPS), consultations with a general practitioner, contact with an association for victims, MSPC initiation and, if not, reasons for not having initiated it. Logistic regressions were used to examine factors associated with MSPC initiation.

**Results:**

Among the 450 respondents, 154 reported having initiated a MSPC after the attacks. Of the 134 who provided the MSPC initiation date, 50% did so during the first month. Among the respondents with at least one of the considered psychological disorders, 53% declared not having initiated yet a MSPC. The primary three reasons for not having initiated a MSPC among people with PTSD were “did not feel the need”, “it was not the right time to talk about it”, and “not offered”. For people with at least one psychological disorder, MSPC initiation was associated with the number of somatic problems, type of exposure (witness, threatened, indirectly exposed), prior psychological treatment, being a woman, being in a relationship, having consulted a psychiatrist or a psychologist, having received an OPS, and being in contact with association for victims.

**Conclusion:**

The organization of adequate psychological care after a terror attack must take into account the need for healthcare that may emerge several months after the attack, and that witnesses seem less likely to receive MSPC than persons directly threatened despite their psychological disorder. Associations for victims and OPS seem to facilitate access to MSPC. Furthermore, our findings highlight the need to train physicians to screen for psychological disorders in persons exposed to terrorist attacks who present with somatic disorders.

**Supplementary Information:**

The online version contains supplementary material available at 10.1186/s13690-023-01206-z.

**Table:** 

Text box 1. Contributions to the literature
• Adapting mental health care provision to the needs of people exposed to terrorist attacks is a public health issue.
• Few studies have focused on the initiation of multiple-session psychological care which is necessary to heal post-traumatic stress disorder and depression.
• This study on psychological care initiation by people exposed to November 13 2015 attacks highlighted important avenues for the organisation of such care: maintaining the mechanisms facilitating access to care over time, not forgetting witnesses, the beneficial role of outreach psychological support and victim associations, raising the awareness of health care workers to identify this need.

## Background

On 13 November 2015, terrorist attacks occurred in the city centre and suburbs of Paris, France. Three suicide bombings took place near a stadium, followed by one suicide bombing and several mass shootings in restaurants in Paris city, as well as a mass slaughter at the Bataclan Theatre. The attacks killed 130 people, injured 643 [1], and exposed several thousands to psychological trauma. After the attacks, emergency psycho-social support units (called “CUMP”) were deployed to provide immediate support to affected communities [2], first in the streets in proximity to the attacks, and later in dedicated information centres set up in town halls of the affected districts. One of the tasks of this field support system was to provide exposed persons with information to facilitate access to psychological care later on if necessary. These centres remained open for one month. Individuals who considered themselves as victims could also contact one of the permanent victim support associations managed by the Ministry of Justice [3] or one of the several non-governmental victims’ associations for social, judiciary or psychological support. But France’s standard healthcare system was responsible for longer-term psychological management. This standard system is based on a combination of public and private facilities. Specialized psychiatric departments in public hospitals provide psychological care for patients at modest consultation fees, reimbursed by the national health insurance system. There are also some public psychological consultation centres dispatched throughout France which offer free psychological consultations (Medical Psychological Centers). In the private sector, psychological care can also be provided in private practice mainly by specialists (psychiatrists or psychologists), but also by general practitioners or psychoanalysts. General practitioners’ consultations have a fixed fee partially reimbursed by health insurance. Psychiatrists’ consultation fees are fixed by the practitioner and can be much higher than the price that the social security reimburses. The visit to a psychologist or a psychoanalyst is not reimbursed. In addition, the French healthcare system allows patients to directly consult a psychiatrist without any referral. At the time of the attack, public psychological care supports were already saturated and the agenda of the GPs very busy under normal circumstances*.* This is why the French Ministry of Health offered free-of-charge consultations for up to two years with a psychiatrist or a psychologist to persons registered as victims by the Ministry of justice [4].

Adequately identifying and meeting the needs of people suffering from psychological consequences of a terror attack and providing them with appropriate treatment and care is a fundamental public health issue [5–7]. A high prevalence of cases of PTSD and depression is observed in persons whose lives are threatened, as well as among direct witnesses to such events and among bereaved persons [8, 9]. Such disorders may negatively impact relationships and work capacity; they can also induce other mental health problems and somatic disorders [10, 11]. Furthermore, many develop PTSD symptoms classified as partial PTSD, to whom providing treatment is increasingly recommended because partial PTSD can also become chronic, associated with other psychiatric disorders, with functional difficulties and with a need for mental health support [12–14]. Effective psychological interventions for persons who present symptoms of such mental health disorders exist [7, 15–17], but cannot be done in an unique session [16, 17]. Lastly, psychological care needs are often wide-ranging, and depend on the person's social and professional situation [16]. Meeting these needs also cannot be done in a single session, and often requires a follow-up.

Although studies on mental healthcare use after a mass traumatic event could provide valuable information to better address mental healthcare needs, the literature on this topic is scarce [18]. In addition, most studies have focused only on whether or not those affected consulted healthcare [6, 19, 20]. It is important to go further, since a one-time consultation is generally not enough. Therefore, the question of initiating a multiple-session psychological care (MSPC) ought to be investigated [6, 21]. Specifically, our research questions were:

- What were the characteristics of persons who initiated a MSPC compared to those who did not, including exposure to the attacks, elevated symptoms of PTSD and depression, demographic characteristics, social support, and history of psychological care?
- Were somatic symptoms associated with initiating a MSPC?
- What was the association between the use of other psychological support, (Outreach psychological supports like CUMP, victim associations, France’s standard healthcare system) and subsequent initiation of a MSPC?
- Given that studies on health literacy highlight that a significant proportion of people with symptoms do not seek appropriate care [1, 7, 19, 22–24], what were the main perceived barriers to MSPC initiation?

In the present paper, we used data from the ESPA 13 November study [1], which was conducted by Santé publique France (French National Agency of Public Health) 8 to 12 months after the November 2015 attacks, to identify factors associated with psychological care initiation among people exposed to the attacks.

## Method

### Recruitment in ESPA survey

The survey *“Etude de Santé Publique post-Attentats du 13 Novembre” (ESPA-Public health Study on Post-13 November Attacks*) is a web-based survey which launched on 7 July 2016 and ended on 10 November 2016. The survey population comprised civilians over 15 years old, whose exposure to the attacks of November 13^th^ 2015 in Paris and its suburbs met criterion A of the DSM-5 definition of PTSD (https://www.ptsd.va.gov/professional/assessment/adult-sr/ptsd-checklist.asp) as follows: persons directly threatened (A1), direct witnesses (A2), persons who had a close one present or killed during the attacks (A3). Civilians who were threatened or were direct witnesses in the police assault against terrorists on November 18th 2015 in the Parisian suburb Saint-Denis were also eligible to participate.

Information about the ESPA survey was disseminated in the media and via an active approach with key stakeholders (CUMP officers, victims, and associations). Furthermore, a study information campaign was complemented with the distribution of letters in mailboxes of residents living in proximity to the places where the attacks took place [1].

### Data collection

**Fig. 1:**
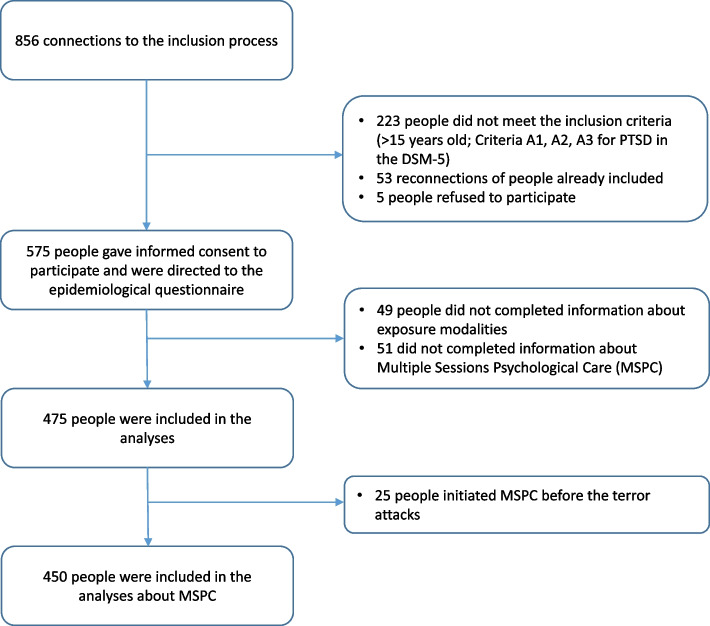
Data flowchart of the MSPC initiation analysis (ESPA 13 November study)

The survey information documents contained web addresses and links where the persons could access the survey questionnaire and get all the information about the survey. If they wished to participate, they were then invited to complete an inclusion questionnaire to verify whether they were eligible to participate. Informed consent was also provided by respondents. Then, the eligible ones were directed to a web-based epidemiological questionnaire. Participation was voluntary. The current analyses concerned the 475 participants who answered the questions about MSPC initiation and about attacks exposure (Fig. 1).

### Variables from the epidemiological questionnaire used for the analysis

Details about the questions asked can be found in Additional file 1.

We collected the following information according to the current level of evidence [25–29] about factors associated with the use of health services:

#### Use of psychological supports

Participants were asked whether due to psychological problems since the attacks they had [1]:

- received an outreach psychological support either provided by professionals in psychology (from CUMP) in the streets immediately after the attacks, during a visit to one of the ad-hoc information centres, at the police station, or at a specific occupational medicine support system which was also set up on an emergency basis.
- consulted a specialist (psychologist or psychiatrist in an hospital, in an unit for the treatment of psycho-trauma, in a Medical-Psychological Center (Centre Médico-Psychologique, CMP), or in a private practice)
- consulted a General Practitioner (GP)
- contacted a member of an association for victims (i.e., Ministry of Justice or NGO).

#### Initiation of a multiple-session psychological care (MSPC)

Participants were asked whether they had initiated a multiple-session psychological care (MSPC) since the attacks (“Since the events, have you initiated a MSPC?”) (yes/no) and on what date [1] ?

Those who answered “no” were asked to give the reason(s) why from the following list: ‘It was not offered to you’, ‘It was offered to you but you did not feel the need’, ‘You did not know it was possible’, ‘You did not want to talk about it/it was not the right time to talk about it’, ‘The arrangements offered did not suit you’, ‘You did not find a professional available’, ‘You had a negative prior experience with a mental health professional’, ‘You were already being provided mental health care before the attacks’, ‘You did not know where to go to receive psychological care’, ‘Financial cost’, and ‘Other reasons, please specify’.

#### Socio-demographics

Information was collected on gender, age, educational level (third level education *versus* upper secondary school certificate (USSC) or less), socio-professional category (Upper-management/Middle-management/Workers-employees-others), employment situation (employed, out-of-work, retired, student.), matrimonial status (in a relationship (‘married/in-a-union/in-a-common-law-relationship’) or not (‘single/divorced/widowed”) [1].

#### Exposure

Exposure was classified into categories: directly threatened (wounded, hit by the blast, targeted by the terrorists, in the indoor or on the terraces of the restaurants attacked or the Bataclan), direct witness (by sight, hearing, or touch), indirectly exposed (having a close one present or killed during the attacks). For some analysis, the exposure variable took into account whether the person was only a witness or was only threatened, or whether he/she had also been indirectly exposed.

#### Psychological history and social isolation

The history of exposure to traumatic situations was assessed by asking participants whether before the attacks, they had been exposed to at least one of listed potentially traumatic situations. Psychological treatment history was assessed by asking participants whether they had been treated for depression or stress for at least six months before the attacks. Participants were asked if they felt isolated (‘quite’ or ‘very alone’) or supported (‘quite’ or ‘very well supported’).

#### Psychological problems

The exposure modalities of all participants met criterion A “having been confronted with death, a threat to life or serious injury as a result of the terrorist attacks of November 13 2015” necessary for the definition of post-traumatic stress disorder (see “recruitment in ESPA survey” paragraph). Post-traumatic stress disorder was measured using the PTSD checklist for DSM-5 (PCL-5) [30]. This scale checks the presence for at least one month (criterion F) of symptoms of intrusion (criterion B: 5 possible symptoms), persistent avoidance of trauma-related stimuli (criterion C: 2 possible symptoms), negative alterations in cognition and mood (criterion D: 7 possible symptoms), hyperarousal (criterion E: 6 possible symptoms). Each response to one of the 20 PCL-5 questions with a score of 2 (i.e. “moderately”) or more on a scale of 0 “not at all” to 4 “extremely” was defined as a present symptom of PTSD. We also asked if the symptoms made it difficult for the person to work, if they had trouble getting along with friends, if relationships with family were more difficult, or if they posed problems for their general level of functioning in life. These four questions were designed to determine whether the person’s situation met DSM-5 criterion G for PTSD (“symptoms create distress or functional impairment”), and to identify people with probable or full PTSD who might need to initiate a MSPC. People with at least one symptom B, one symptom C, two symptoms D and two symptoms E were considered likely to have a full PTSD. If at least two of the criteria B, C, D, E were met, the person was considered as probably suffering from partial PTSD, as proposed in McLaughlin’s study based on WHO mental health surveys [13].

The Hospital Anxiety and Depression Scale (HADS) [31] was used to identify a probable depressive disorder that warranted clinical assessment and management (score ≥ 8 on the depression (HAD-d) subscale [32]. As the symptoms collected by the HAD-a could as well be elevated in case of symptoms of post-traumatic stress, we preferred measuring the presence of probable partial PTSD defined as recommended by McLaughlin [14].

We considered that persons who satisfied one of the previous definitions were most likely to have needed the initiation of a MSPC.

#### Somatic problems

Participants were asked whether they had experienced the following somatic problems at least once since the event: headaches or migraine, osteoarticular problems or back pain, stomach aches or spasmodic colic, asthma or breathing problems, gastric ulcers or stomach pain, high blood pressure, dermatological problems, unbalanced diabetes, cardiac problems, fatigue or weariness, concentration problems, sleep disorders, and tinnitus [33]. They were also asked whether they considered at least one of these problems linked to the event and whether they had spoken to a doctor about the problem. The variable ‘somatic problems’ summed the number of different problems declared by the participant.

### Statistical analyses

We first explored the distribution of dates of MSPC initiation. The proportions for each of the reasons given for not seeking MSPC were calculated for persons with PTSD, partial PTSD or depressive disorders (pooled together for sample size reasons), and for those with none of these conditions. Based on the literature [25–29], we computed univariate logistic regressions for participants with at least one of these disorders to test for an association between having initiated a MSPC or not and socio-demographic characteristics, exposure type, psychological treatment history, social isolation, somatic problems, having received an OPS, consultation with a GP, consultation with a specialist (psychiatrist or a psychologist), and contact with a member of an association for victims. Based on the significant results of the univariate analysis and if not on the literature for some variables, we then tested multivariate models to explain MSPC initiation. In model 1 we adjusted for socio-demographic variables (gender, matrimonial status and educational level), exposure level, the number of declared somatic problems, history of trauma, psychological treatment history and social isolation. In model 2 we added the healthcare-variables “having received an OPS”, “consultation with a GP”, and “contact with a member of an association for victims”. We considered it relevant to have a separate model without the healthcare variables, since they might be potential facilitating factors as well as important steps towards MSPC. Finally, in model 3 we additionally included the variable “consultation with a specialist”. We added this variable separately, as it could potentially be part of the MSPC. Analyses were performed using SAS Enterprise Guide 7.11.

### Ethics

When answering the online questionnaire, participants had access to a telephone line (10 am-10 pm, 6/7 days) if they needed support from trained psychologists. The study was approved by the Commission Nationale Informatique et Libertés (CNIL, National Commission for Data Protection and Liberties, authorization demand n°915262v2, deliberation n°2016–209 of 7/2016) and a Committee for the Protection of Persons (amendment number 7035/3/3283). Visitors to the website had access to information on possible consequences of exposure to these attacks and on how to seek care. Respondents under 18 years old had to provide authorization from a parent to participate.

## Results

### Respondents’ characteristics (Table 1)

**Table 1:** Demographics, exposure type, and use of mental health support services, ESPA 13 November Survey, *N* = 475

	N	Mean
**Age (mean)**	473	39,8
		SD: 12.5
**Mean number of different somatic symptoms**	441	4.0
		SD: 2.6
	N	%
**Exposure**	475	
Threatened only	65	14
Witness only	145	31
Indirectly exposed only	96	20
Threatened *and* indirectly exposed	96	20
Witness *and* indirectly exposed	73	15
**PTSD**	465	
No	172	37
Partial	115	25
Full	178	38
**Probable depression HAD-d ≥ 8**	471	
No	320	68
Yes	151	32
**Probable depression ** ***or partial or full*** ** PTSD**	467	
No	165	35
Yes	302	65
**Gender**	473	
Woman	316	67
Man	157	33
**Educational level**	475	
Upper secondary school certificate (USSC) or less	85	18
Higher than USSC	390	82
**Socio-professional category**	470	
^a^Upper and middle management	290	62
^b^Workers, employees, others	180	38
**Professional situation**	475	
Professionally active	373	79%
Student	41	9%
Retired, full-time home maker	34	7%
Unemployed	27	6%
**Matrimonial situation**	475	
Married, in a civil union, common-law relationship	258	54
Single, divorced or widowed	217	46
**Psychological treatment history**	473	
Depression with and without stress	88	19
No	385	81
**History of trauma**	470	
No	311	66
Yes	159	34
**Social isolation**	471	
Felt supported	351	75
Felt isolated	120	25
**Outreach psychological support**	475	
No	304	64
Yes	171	36
**Victims’ or Victims’ sup**port Association	475	
No	404	85
Yes	71	15
**Visit to general practitioner**	475	
No	396	83
Yes	79	17
**MSPC initiation after 13/11/2015**	450^c^	
No	296	66
Yes	154	34

^a^Upper and middle management: craftsperson, trader, business leader, professor, senior intellectual

^b^Workers, employees, other: employee, blue-collar worker, technician, no professional activity, other

^c^Among the 475 respondents 25 had already a MSPC at the time of the terrorist attacks

Most respondents were women (67%), middle-aged (40 years), well educated (82% > upper secondary school certificate USSC), with a professional position of upper or middle manager (62%) and professionally active (79%). Persons who were only directly threatened, only witnesses, or only indirectly exposed accounted for 14%, 31%, and 20% of the study population, respectively. Directly threatened individuals and witnesses who had also been indirectly exposed accounted for 20% and 15%, respectively.

In terms of mental disorders, 37%, 25%, and 32% had probable PTSD, partial PTSD and probable depression (HAD-d ≥ 8), respectively. Overall, 65% of the sample was screened as having probably at least one of these disorders.

### MSPC initiation

Among the 475 respondents, 25 (5%) declared they were already receiving MSPC before the attacks and were excluded from the present analyses. Of the remaining 450, 34% (*n* = 154) declared having initiated a MSPC after the attacks.

**Fig. 2:**
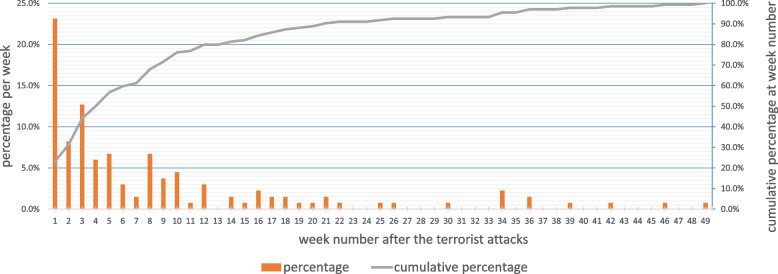
Distribution of MSPC initiation time in civilian respondents (ESPA 13 November Study) *n* = 134

Of the 134 persons who initiated a MSPC after the attacks and provided an initiation date, 80% did so in the first three months (50%, 18% and 12% in the first, second and third months, respectively (Fig. 2). At the time of the survey, among the persons who had declared having initiated the MSPC during the first month, 77% were still receiving it.

### Reported reasons for not initiating a MSPC

**Fig. 3:**
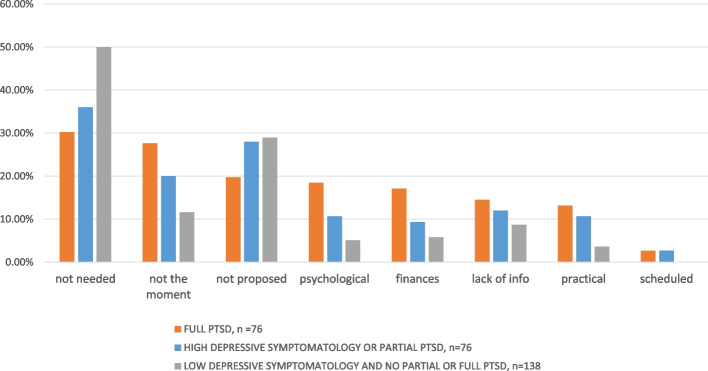
Distribution of expressed reasons for not initiating a MSPC in civilians *n* = 296 (ESPA 13 November Study). not needed: ‘It was offered to you but you did not feel the need’, mentioned in ‘Other reasons’. not proposed: ‘It was not offered to you’. not the moment: ‘You did not want to talk about it/it was not the right time to talk about it’. lack of info: ‘You did not know it was possible’, ‘You did not know where to go to receive psychological care’. finances: ‘Financial cost’. psychological reasons: ‘You did not want to talk about it/it was not the right time to talk about it’, ‘You had a negative experience of a former contact with a mental health professional’, ‘Other reasons: previous perceived unprofessional conduct of a doctor, feeling you did not deserve treatment, wanting to cope without treatment. felt inhibited to initiate. practical reasons: ‘The arrangements offered did not suit you’, ‘You did not find a professional available’, and ‘Other reasons: ‘ geographical distance’, ‘difficulties leaving apartment/house’’, ‘going to treatment has become complicated because of a change in job/work’. scheduled: when mentioned in ‘Other reasons, please specify’

Among those with probable full PTSD (Fig. 3), the most common reasons given for not initiating a MSPC were ‘It was offered to you but you did not feel the need’ (30%), followed by ‘You did not want to talk about it / it was not the right time to talk about it’ (28%) and ‘It was not offered to you’ (20%).

The same three reasons topped answers for participants with only probable depressive disorders or probable partial PTSD, but in a different order. Specifically, 36% did not feel the need, 28% reported not having been offered treatment, while 20% replied they didn’t want to talk about it/it was not the right time.

Again, these reasons were prominent among those with none of these three disorders. Specifically, 50% did not feel the need, 29% had not been offered MSPC, and 12% reported they did not want to talk about it/it was not the right time.

Among persons with no probable full PTSD, the proportions for other reasons were clearly lower. In contrast, in those with probable full PTSD, 18% of respondents also frequently mentioned reasons which we grouped and labelled under ‘psychological reasons’, and 17% mentioned financial worries. Finally, 14% of this group said they lacked ‘information’ (Fig. 3), while 13% mentioned practical reasons (Fig. 3).

### Characteristics associated with MSPC initiation in civilians with at least one mental health disorder

Among the 287 respondents suffering from probable partial or full PTSD, or probable depressive disorder and who didn’t have initiated MSPC before, respectively 136/151 (47%/53%) declared having initiated/not initiated a MSPC after the attacks.

#### Univariate models (Table 2)

**Table 2:** Univariate analysis of factors associated with MSPC, civilians with HAD-d ≥ 8, partial or full PTSD (ESPA 13 November, *n* = 287)

**Logistic regression**		**Univariate logistic regression**			
**Independent Variables**		**MSPC Initiation**			
	**Reference category**	**OR**	**95% CI**		***P***
**Number of reported somatic problems**		1.25	1.12	1.39	< .0001
**Exposure**					
Threatened only	Witness only	6.17	2.62	14.54	< .0001
Indirectly exposed only	Witness only	2.62	1.20	5.71	0.015
Threatened and indirectly exposed	Witness only	8.05	3.64	17.81	< .0001
Witness and indirectly exposed	Witness only	2.31	0.96	5.57	0.061
**Age**		0.99	0.98	1.01	0.547
**Socio-professional category**					
Upper and middle management^a^	Workers, employees, other^b^	0.98	0.61	1.56	0.637
**Professional situation**					
Professionally inactive^c^	Professionally active	0.88	0.53	1.50	0.631
**Gender**					
Woman	Man	1.55	0.92	2.59	0.097
**Level of education**					
USSC or less	Higher than USSC	1.04	0.59	1.834	0.900
**Matrimonial status**					
Married, cohabiting, Civil union	Single, divorced, widowed	1.75	1.09	2.81	0.020
**Social isolation**					
Felt supported	Felt isolated	1.04	0.63	1.71	0.881
**History of trauma**					
Yes	No	1.06	0.65	1.71	0.826
**Psychological treatment history**					
**Yes**	No	1.91	1.08	3.38	0.026
**Outreach Psychological Support**					
Yes	No	2.08	1.29	3.35	0.003
**Visited a GP**					
Yes	No	2.57	1.45	4.56	0.001
**Support from an association for victims**					
Yes	No	5.43	2.78	10.61	< 0.001

^a^Upper and middle management: craftsperson, trader, business leader, professor, senior intellectual

^b^Workers, employees, others: employee, blue-collar worker, technician, no professional activity, other

^c^Student, retired, full-time home maker, unemployed

The likelihood of initiating a MSPC was positively associated with the number of declared somatic problems, with all the exposure modalities compared to being witness only (being threatened only, being indirectly exposed only, being threatened and also indirectly exposed, being witness and also indirectly exposed), with having a history of psychological treatment, with being in a relationship, with having benefited from OPS, with having for psychological difficulties visited their GP, consulted a specialist or contacted an association for victims.

MSPC initiation was not associated with the socio-professional category, the professional situation, the age, gender, or education level, neither with the perception of social isolation.

Odds ratios and confidence intervals are reported in Table 2.

#### Multivariate models (Table 3)

**Table 3:** Multivariate analysis of factors associated with MSPC, civilians with HAD-d ≥ 8, partial or full PTSD (ESPA 13 November, *n* = 287)

**Logistic regression**		**Model 1**				**Model 2**				**Model 3**			
**Independent Variables**		**MSPC initiation**				**MSPC initiation**				**MSPC initiation**			
	**Reference category**	**OR**	**95% CI**		***P***	**OR**	**95% CI**		***P***	**OR**	**95% CI**		***P***
**Number of reported somatic problems**		1.20	1.06	1.35	0.004	1.19	1.05	1.35	0.007	1.20	1.05	1.37	0.006
**Exposure**													
Threatened only	Witness only	7.25	2.73	19.25	< .0001	4.17	1.47	11.87	0.008	3.18	1.07	9.46	0.037
Indirectly exposed only	Witness only	2.57	1.08	6.13	0.034	2.04	0.80	5.17	0.135	1.72	0.65	4.56	0.279
Threatened and indirectly exposed	Witness only	10.72	4.21	27.28	< .0001	7.35	2.73	19.75	< .0001	4.28	1.51	12.18	0.006
Witness and indirectly exposed	Witness only	3.11	1.16	8.32	0.024	3.17	1.15	8.76	0.026	3.27	1.15	9.33	0.027
**Gender**													
Woman	Man	2.51	1.33	4.73	0.005	2.77	1.41	5.42	0.003	2.89	1.43	5.82	0.003
**Level of education**													
USSC or less	Higher than USSC	0.91	0.46	1.81	0.786	0.64	0.31	1.32	0.226	0.59	0.28	1.26	0.172
**Matrimonial status**													
Married, cohabiting, Civil union	Single, divorced, widowed	2.06	1.16	3.67	0.014	2.37	1.28	4.38	0.006	2.67	1.40	5.11	0.003
**Social isolation**													
Felt supported	Felt isolated	1.32	0.71	2.46	0.380	1.05	0.54	2.04	0.880	0.95	0.48	1.89	0.876
**History of trauma**													
Yes	No	1.22	0.67	2.20	0.514	1.42	0.76	2.64	0.270	1.53	0.80	2.92	0.200
**Psychological treatment history**													
**Yes**	No	2.36	1.20	4.62	0.012	2.70	1.30	5.61	0.008	2.65	1.23	5.72	0.013
**Outreach Psychological Support**													
Yes	No	X	X	X	X	2.04	1.11	3.77	0.022	2.14	1.14	4.03	0.018
**Visited a GP**													
Yes	No	X	X	X	X	1.50	0.73	3.08	0.268	1.24	0.59	2.58	0.575
**Support from an association for victims**													
Yes	No	X	X	X	X	5.17	2.29	11.66	< .0001	8.05	3.32	19.48	< .0001
**Consulted a specialist**													
Yes	No	X	X	X	X	X	X	X	X	3.60	1.86	6.94	< .0001

After the multivariable adjustments in model 1, the likelihood of initiating a MSPC became significantly associated with female gender. Furthermore, initiation of a MSPC remained associated with the number of declared somatic problems, with all the exposure modalities compared to being witness only (being threatened only, being indirectly exposed only, being threatened and also indirectly exposed, being witness and also indirectly exposed), with having a history of psychological treatment and with being in a relationship. After the addition of the healthcare variables in model 2, being indirectly exposed only was no longer significantly associated with MSPC initiation. Furthermore, having benefited from OPS and having contacted an association for victims were significantly associated with MSPC initiation, but not GP visits. Otherwise there were no changes in terms of the variables significantly associated with MSPC initiation compared to model 1. When we added consultation with a specialist in model 3, the latter was also associated with MSPC initiation.

## Discussion

In our study sample, which gathered the highest number of civilian adults meeting criterion A of the DSM-5 definition for PTSD [1] of all French studies on the impact of terrorist attacks to date, 66% of all respondents, and 53% of those suffering from probable partial or full PTSD or depression, did not initiate MSPC after the attacks. The most frequent reason given for this was a perceived lack of need. This finding corroborates results from studies conducted with people with severe mental health problems both in everyday life [34] and in the aftermath of a terror attack [19]. The psychological reasons for not initiating a MSPC reflected those often cited in literature [19, 35, 36]. In terms of prevention, this finding highlights the need to help persons to balance these feelings with the perceived benefits of engaging in a MSPC. Another reason frequently mentioned for not initiating a MSPC was that “it was not the right time to talk about what happened”. This underlines the importance of providing victims with sustainable comprehensive information and treatment options, which can be adapted to changing perceived care needs over time [37]. Indeed, some victims put on the back burner the need for psychological treatment of trauma as long as the needs for information and reorganisation of daily life disrupted by exposure to the event are not met. Moreover, post-traumatic stress disorders may appear late in some people and it is a chronic condition that may last for several months or years. The financial aspect in the decision to initiate MSPC was mentioned quite frequently (17%) by those who had PTSD related to the attacks. This result justifies the initiative of the French Ministry of Health, which offered free-of-charge consultations with a specialist to persons registered as victims. Finally, difficulty of access to information and to care were also cited reasons for no MSPC initiation, but to a lesser extent.

Among those suffering from probable partial or full PTSD, or probable depressive disorder, our study also showed an association between MSPC initiation and modalities of exposure to the attacks. This is in line with the association between contact with a mental health service and exposure found in other studies on terrorist attacks [6, 21]. In our study, witnesses with full/partial probable PTSD and/or depression were less likely to initiate MSPC than persons who were threatened and had one or more of these disorders. This may be the result of perceived legitimacy to receive care on the part of the witnesses, as well as health care policy on the part of authorities, which focused on more direct victims.

We found that the initiation of a MSPC was associated with somatic problems. In a study by Holman et al*.* [38], the stress caused by exposure to the 9/11 attacks increased the frequency of somatic disorders by 18% and led to greater utilisation of mental health care. Stuber et al*.* highlighted that people with physical health problems after the 9/11 attacks were more likely to have sought mental health services [19]. After the attack on Utoya Island, the cumulative somatic problems score predicted the use of specialist mental health care [5]. Somatic problems may strengthen links with the health care network and therefore increase the likelihood of seeking mental health care specialist [5]. Somatic problems contribute also to diminished functioning and could maintain mental illness at long-term [5]. They deserve a special attention as an alert for psychological problems.

A history of treatment for depression or stress prior to the November 2015 attacks was associated with the initiation of a MSPC in our study. Stuber et al. found that six months after the 9/11 attacks [19], the use of mental health services by New Yorkers was clearly linked to prior contact with the mental health care system and with knowledge of how it works.

In the literature, woman gender is associated with a greater risk of PTSD after exposure to terrorist attacks [39]. The influence of gender on the use of mental health care is less clear. Although some post-attack studies showed no differences in gender by mental health care uptake [6, 19, 20, 40], others showed greater use by women [5, 21, 37, 41]. However, in multivariate models, this difference remained significant in only one study [41]. Among the relatives of the Utoya island victims, analysis of health care registries showed an increase in the use of specialist mental health care only in women, while visiting GPs increased in both genders [42]. In our multivariate models, gender was significant in terms of MSPC initiation, which suggests that among persons with probable partial or full PTSD or probable depressive disorders, an increased probability of initiation of a MSPC after terrorist attacks is associated with being a woman.

Having a GP was associated with accessing mental health care in a general population study after the 9/11 attacks [38]. GPs are considered the ‘expected’ care route to consultation with a specialist [7], although their role may vary according to the health system and the specific context [1]. In our study, visiting a GP was associated with MSPC initiation in univariate analysis but not in multivariate analysis; this suggests that it is not a strong determinant of MSPC initiation, given other characteristics of the population studied. On the contrary, visits to a specialist were strongly associated with the initiation of a MSPC. This may reflect a tendency for victims to use a specialist for their psychological problems rather than a general practitioner. This propensity may have been reinforced by a free-of-charge specialist consultation policy for victims of terrorist attacks in France. It may also reflect that specialists recognize more easily the indications for psychological care than GPs. Nevertheless, it could also be that the respondent declared their first session of a MSPC as a “visit to a specialist”.

The fact that psychological care initiation was significantly associated with OPS and contact with associations for victims after the multivariable adjustments in model 2 and 3, suggests that the field-based consultations and healthcare information provided by OPS as well as the collective support framework provided by associations for victims may facilitate access to psychological care in case of needs. For example, the members of the CUMPs who provided consultations in the support and information centers set up near the sites of the attacks, informed patients about the symptoms they might be experiencing as a result of the attacks and distributed a document containing the addresses of places where they could obtain psychological support.

In our models, a feeling of social isolation was not associated with MSPC initiation. In other studies, while the perceived quality of social support was inversely associated with the intensity of psychological symptoms experienced [23] and negatively correlated with feelings that care needs were unmet [23, 29], the influence of social support on seeking care was less clear. On the one hand, this support can reduce the intensity of symptoms and the need to seek care [43], in turn reducing the propensity of initiating a MSPC. On the other hand, it can facilitate the sharing of information and access to care [43]. The association that we found between being in a relationship and initiating a MSPC may be a marker for the role that a partner plays in supporting their mate to initiate mental health care.

Some post-attack studies have shown that people under 65 years of age were more likely to use mental health care [41], and that low level of education was associated with less use of care in times of need [21]. The lack of influence of age and education on MSPC initiation in our study may be due to the homogeneity of the Parisian population exposed to the attacks: a majority were middle-aged, well educated, and with an intermediate to high professional status. Moreover, access to healthcare in Paris is better than in other areas of France [1]. This could mitigate the influence of the above-mentioned social demographic variables on access to care.

Eight to 12 months after the November 2015 terror attacks in Paris and its suburbs, 34% of study’s participants who didn’t have already a MSPC at the time of the attacks, had initiated a MSPC. During the first month, the Nice and TENTS guidelines [17, 44–46] for post-disaster psychosocial care underscore the importance of first and foremost promoting social support, and reserving mental health care interventions to prevent PTSD for specific clinical indications (e.g., acute stress disorder) for which there is already evidence of intervention effectiveness [44]. In our study, 50% of MSPC initiation occurred within the first month. This result suggests that an important proportion of the people exposed felt a need for psychological care with a follow-up before PTSD or depression had time to appear.

Our results should be interpreted keeping in mind the specificities of the survey methodology used.

We did not have access to the Ministry of Justice’s list of victims (itself non-exhaustive) [47] in order to compare it with our list of participants, or to calculate the participation rate. The eligible persons were of course free of responding or no to the survey. Therefore, there may have been differences between terror-exposed individuals who participated in the study and those who did not, which may have introduced selection bias. Given that the study took place just a few months after the attacks, those suffering the most may have felt that it was too difficult to participate [36]. On the other hand, those suffering less may have felt less motivated, or that their participation would be less legitimate. Furthermore, our web-based survey excluded people who had no internet access and those most socially disadvantaged [36]. Nevertheless, the Parisian population affected by the attacks was essentially middle-aged, active, educated and with access to the Internet.

Our web-based questionnaire may have encouraged higher response rates to questions on sensitive topics compared to face-to-face interviews [48]. We used validated scales for screening the main mental disorders examined, with expected good sensitivity and specificity [30, 32]. But as it was web-based, there was no clinical examination, which is the reference diagnostic method. This may have resulted in inaccuracy in our diagnoses. The HAD_d scale measures depressive symptom scores but does not diagnose directly any specific depressive disorder that would warrant MSPC initiation. Although, studies on various populations have established associations between these scores and the likelihood of presenting depressive disorders, results of evaluations of the HAD_d for screening for possible depressive disorders among civilians exposed to terrorist attacks are particularly relevant. The study on the psychological impact of the January 2015 terrorist attacks in the Paris Region [49] which used simultaneously the HAD_d and face-to-face interview with the Mini International Neuropsychiatric Interview (Mini) underlined satisfactory performances of the HAD_d to screen for depression disorder among the exposed civilians with an optimal threshold score of 7.5 [50]. That being said, although these scales measure the intensity of symptoms, they may not sufficiently assess the presence of functional impairment, which is the main trigger for subsequent care [51]. For this reason our study considered the negative impact of PTSD symptoms on everyday life (criterion G “functional significance”), in addition to DSM-5 criteria A, B, C, D, E and F.

As data in our study were collected from participants’ self-reports, our results are subject to recall bias about care consumption [36]. However, this bias is more important concerning the number of visits rather than the declaration of using or not the different types of medico-psychological cares available [52].

The socio-demographic and health care network specificities of the Paris region may make it difficult to extrapolate the results to the whole of France in terms of MSPC initiation. The external validity of the findings to other countries may also depend on their health systems and plans for post-disaster psychosocial care.

Finally, the cross-sectional nature of the study prevented us from assessing whether the observed correlations might be causal.

Our method of collecting information gives only an instantaneous view of the person’s state of health at the time of responding and our analysis compared it to the initiation of a psychological care at any time after the exposure. Yet, while PTSD tends to be chronic in the absence of treatment, it is typical for individuals to experience fluctuating symptoms, including remission and reappearance of symptoms over time [53]. It is known that a proportion of people with PTSD will recover from the disorder within a few months, that another proportion will not recover without treatment, and that a small proportion may not even develop the disorder until several months after the exposure [53]. The time elapsed between exposure and participation in the study may have influenced the prevalence of the disorders studied. However, events considered as intentional (like terrorist attacks) are associated with greater persistence of PTSD symptoms than when the event is unintentional [54]. Studying the impact of the attack 8 to 11 months after the exposure is therefore not too late to measure its impact.

The psychological consequences of trauma exposure tend to be more severe and disabling when they result from interpersonal violence [53]. The high level of disability, mental and physical co-morbidity and loss of quality of life experienced by these people, and the significant personal and collective costs resulting from the social consequences of the disability (housing problems, absenteeism and unemployment) [55], argue for the development of collective strategies to identify people with disabling mental health disorders at an early stage, so that they can be offered access to appropriate care if they so wish. A great deal of research remains to be done to gain a better understanding of the factors that predict different trajectories, and to develop more effective strategies for screening and providing psychological care to people exposed to attacks at the right time [16]. Addressing all aspects of this field requires numerous studies. In the opinion of the psychologists and first-aid psychiatrists who helped us construct the questionnaire, it was unrealistic to expect all patients to identify and be able to name precisely the type of psychotherapy they received. However, it was reasonable to expect respondents to identify whether or not they had undergone a multiple-session psychological treatment, as opposed to nothing or a one-time consultation. Despite its limitations, this information contributes to building knowledge about to what extent psychological care was provided to those with probable mental health problems in the wake of the terrorist attacks.

## Conclusion

The results of this study highlight that the organisation of psychological care for people exposed to terror attacks should pay special attention to the following facts. First, not only the victims directly threatened but also witnesses must get consideration, since some of them may suffer just as much from post-traumatic pathologies. This is a public health issue considering the size of that population and the difficulties to reach them. Second, for victims, the need for MSPC is not necessarily immediate after an attack but may present itself several months later. It is important to plan sustainable and comprehensive information and treatment options which can be adapted to changing perceived care needs over time. Third, somatic complaints which may lead victims to visit a doctor can also be a sign of psychological suffering. All physicians and even all health professionals who treat victims of attacks should be trained to identify psychological needs. Finally, OPS and associations for victims seem to facilitate MSPC initiation among the persons who present probable disorders after having been exposed to a terrorist attack.

### Supplementary Information

**Additional file 1.** Relevant sections used in the civilian version of the French web-based questionnaire for Phase 1 of the ESPA_ 13_ November study. Additional file 1 is a French language clean-copy of the relevant sections of the web-interview guide used as part of the present study’s design (these sections dealt with the person’s current social and demographic situation; the ways in which the person was exposed to aggression, physical injuries, the loss or exposure of a loved one, the psychological consequences of this exposure, the different aspects of related psychological care, the health consequences other than psychological, the history of exposure to other traumatic exposures, the psychological treatment history, the perceived social support.

## Data Availability

The data for this study are available on request from *Santé Publique France*, but restrictions apply. More specifically, some data were used under license and so are not publicly available. Other data are however available from the authors upon reasonable request and with the permission of *Santé publique France*. The protocol and questionnaire of the survey are available on this DOI link 10.6084/m9.figshare.20552292.

## Abbreviations

ANR: French National Research Agency
CNIL: C*ommission Nationale de l’Informatique et des Libertés*
CMP: *Medical-Psychological Center*
DSM-5: Diagnostic and Statistical Manual of Mental Disorders, fifth edition
ESPA 13 November survey: (*Enquête de Santé Publique post-Attentats du 13 Novembre* = Public health Study on Post-13 November Attacks is part of
CUMP: Emergency psycho-social support unit
GP: General practitioner
HAD: Hospital Anxiety and Depression scale, anxiety subscale (HAD-a) or depression subscale (HAD-d)
MINI: Mini International Neuropsychiatric Interview
MSPC: Multiple-session psychological care
OPS: Outreach psychological support
PCL-5: DSM-5 checklist for PTSD
PTSD: Post-traumatic Stress Disorder
*USSC*: Upper secondary school certificate
